# Unique Considerations in Caring for Rural Patients with Rectal Cancer: A Scoping Review of the Literature from the USA and Canada

**DOI:** 10.3390/jcm14124106

**Published:** 2025-06-10

**Authors:** Lydia Manela Rafferty, Bailey K. Hilty Chu, Fergal Fleming

**Affiliations:** 1 Department of Surgery, Bassett Medical Center, Cooperstown, NY 13326, USA; 2 Surgical Health Outcomes and Reaching for Equity (SHORE), Department of Surgery, University of Rochester Medical Center, Rochester, NY 14642, USA

**Keywords:** rectal cancer, rural health, healthcare access, oncologic outcomes, patient preferences, quality of care, cost of treatment

## Abstract

**Background:** Rural patients, including those with rectal cancer, continue to be underrepresented in research and medically underserved with unique challenges to accessing care. Like the rest of America, rural patients are experiencing rising rates of rectal cancer; however, unlike the rest of the country, they also have rising rectal cancer-related mortality. This study aims to review the literature regarding care for patients with rectal cancer in rural settings, from presentation and diagnosis to treatment algorithms, oncologic outcomes, their unique preferences, and the goals of care. **Methods:** A literature search was performed on PubMed, on 31 October 2024, using synonyms of “rural” and “rectal cancer” to identify relevant articles. Articles from outside the USA and Canada and those offering only commentary were eliminated during the initial screening/retrieval. A full-text review was performed on the remaining articles; all the studies that did not address the identified primary or secondary outcomes in rural rectal cancer patients were then excluded. All the primary and secondary outcomes are briefly summarized in narrative form, with more detail on the primary outcomes provided in tables. The variability in the key criteria between the studies is also summarized in the tables and appendices provided. **Results:** Thirty studies were identified that addressed the outcomes of interest in rural rectal cancer patient populations. The total number of participants could not be assessed given the use of overlapping databases. Of the articles, 21 addressed treatment modalities (surgery, chemotherapy, radiation), 13 addressed oncologic outcomes, and a mix of additional studies addressed the diagnostic work up, costs, and patient preferences. The studies addressing treatment demonstrated similar practices in regard to chemotherapy and surgical management, aside from lower rates of minimally invasive surgery, along with decreased neoadjuvant radiotherapy use and increased under-dosing in rural patients. The oncologic outcomes were overall similar to worse for rural patients as compared to urban patients, even for those receiving treatment at high-volume urban centers. Additionally, rural patients have higher healthcare costs for rectal cancer care. **Discussion/Conclusions:** Rural patients are an at-risk group, with a rising disease burden and worsening rectal cancer outcomes, despite advances in rectal cancer care and improving oncologic outcomes in the general population. Analysis of the situation is complicated due to the underrepresentation of rural patients in research and the lack of uniformity in the definition of “rural”. Moreover, significant gaps in the literature remain, such that the evaluation of guideline-concordant care is incomplete, including an absence of literature about watch-and-wait approaches in rural populations. While regionalization of rectal cancer care has shown promise, the improvements in outcomes may not be commensurate for rural patients. Thus, a specific focus on the impact of this shift for rural patients is necessary to mitigate unintended consequences.

## 1. Introduction

**Figure 1:**
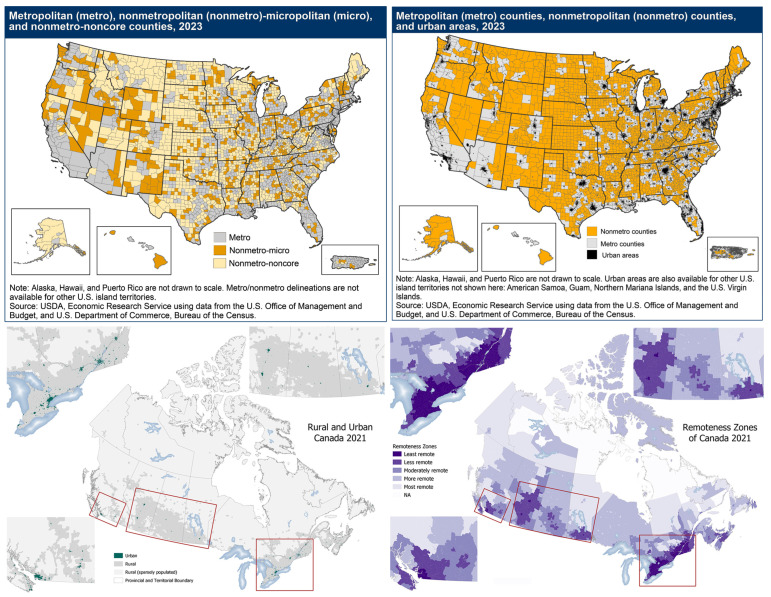
Heat maps of rurality in the USA and Canada, using different measurement metrics [4,5]. This data, published by the USDA and Statistics Canada, helps illustrate the variability in terms of what is defined as rural and serves as a visual guide to where those locations are within each country.

Rectal cancer is a growing problem, with both a rising incidence of colorectal cancer and data demonstrating that rectal cancers comprise a higher percentage of colorectal cancers: up to 31% in 2019 from 27% in 1995 [1]. This trend is especially notable in younger populations, with a steep rise in the incidence of early onset rectal cancer in patients less than 50 years old [2]. This is compounded in rural patients, who, between 2000 and 2016, experienced a 56% increase in early onset rectal cancers, twice that observed in urban populations [3]. While the rural populations in the United States (US) and Canada have generally decreased over the last century, they continue to represent a significant proportion of the population: as of the 2020 census, ~20% of the US population (~66.7 million people) lived in rural areas and 71–97% of the land area of the US is estimated to be rural (Figure 1; [4,5,6,7]). Care for patients in rural areas presents unique challenges divergent from those in urban areas, including disproportionate impacts of the social determinants of health, including higher poverty rates, lower incomes, difficulties in accessing care, and limitations to our understanding of how to best provide care given the persistent underrepresentation of rural patients in clinical trials [8,9,10]. While the last 25 years have produced significant advancements in rectal cancer care, it is unclear whether patients in rural areas are seeing the benefits that these advancements have brought to their urban counterparts.

Treatment for rectal cancer has undergone significant evolution over the last two decades, resulting in the use of the current algorithms that increasingly utilize neoadjuvant therapies and emphasize organ preservation, with the omission of surgery for patients who have a sustained clinical complete response (cCR) to neoadjuvant therapy [11,12,13,14,15,16,17,18,19,20]. Alongside these changes, there has been a movement to shift care for rectal cancer away from community hospitals and toward specialized regional centers. A growing body of literature demonstrates a strong association between being treated at a high-volume hospital (HVH) or by a high-volume surgeon (HVS) and improved oncologic outcomes for rectal cancer patients [21,22,23]. Further research has also shown that the treatment of colorectal cancer by colorectal surgeons (CRSs) decreases costs at both a healthcare system and societal level [24]. This has driven the push for greater regionalization, with the assumption that patients will fare better if directed to HVHs, irrespective of the distance traveled. However, little focus has been given to the impact of these policies on the rural patient population.

Studying rectal cancer in this vulnerable population is challenging given the wide variability in who is classified as “rural” and the lack of a unified standard for defining rurality, both in the literature (Table 1) and by governing bodies [6,7,25,26,27]. However, given the growing burden of disease in this population, while care is increasingly shifting to urban centers, expanding our understanding is critical. This review aims to synthesize contemporary practices in the treatment of rectal cancer in rural patients and their subsequent oncologic outcomes, the unique challenges they face in regard to their care, and their care preferences.

## 2. Materials and Methods

This scoping review on the treatment of rectal cancer in rural patient populations in the US and Canada follows the PRISMA-ScR guidelines. We limited the search to studies from 2000 to 2024 to focus on contemporary rectal cancer management. As this is a secondary analysis of previously published data, this study did not require review by the Research Subjects Review Board.

### 2.1. Search Strategy

With the assistance of experienced medical librarians, a literature search of PubMed, using synonyms of “rectal cancer” and “rural”, was performed on 31 October 2024 to identify all the studies published between 2000 and 2024 that addressed rectal cancer in rural patient populations (Appendix B).

### 2.2. Study Selection

**Figure 2:**
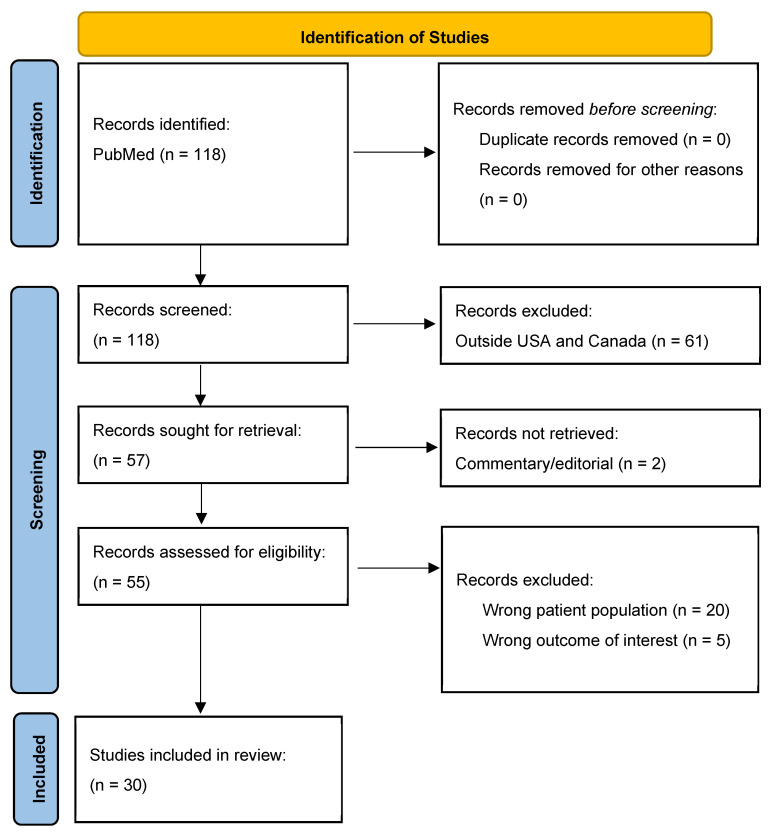
Study selection. A flow diagram of the studies from the MeSH search that were identified, screened, and included vs. excluded from our final analysis.

One reviewer (LR) reviewed all the citations for eligibility. Single center, multicenter, and database studies were included. We screened and excluded all the studies conducted outside the US and Canada. After retrieval, we excluded non-research manuscripts (e.g., narratives, case reports, case series, letters to the editor, etc.). A full-text review was then performed. Studies on the wrong patient population (studies that combined colon and rectal cancer patients, studies that only addressed rectosigmoid cancers, and studies that did not specifically address rural patients with rectal cancer) were excluded. Additionally, studies that did not report on at least one of the outcomes of interest (stage at diagnosis/tumor characteristics, work up, treatment, treatment response, oncologic outcomes, costs of treatment, or care preferences of rural rectal cancer patients) were excluded (Figure 2). Any studies that did not clearly fall into the inclusion or exclusion criteria were discussed amongst the authors until a consensus was achieved.

### 2.3. Definitions

A broad definition of “rural” was used for the inclusion of studies in this review: any studies evaluating an exclusively rural population by self-reporting, controlling for rurality, using a widely accepted instrument (e.g., RUCA or RUCC; [6,7,25,26]), when assessing the primary/secondary outcomes, or explicitly assessing the effects of rurality on rectal cancer patients (even if doing so through the use of an instrument of their own design) were included. Studies using a non-standard metric for rurality that did not explicitly state that they were doing so to try to capture the effects of rurality were excluded.

### 2.4. Data Extraction

A single reviewer (LR) collected the data from all the eligible studies. Variables of interest included the country of origin, year, data source, metric for assessing rurality, inclusion and exclusion criteria, and primary and secondary outcomes. The primary outcomes of interest were used to evaluate the treatment modalities received by rural rectal cancer patients and their oncologic outcomes. Regarding the treatment modality, any studies addressing surgical treatment (including type, timing, and palliative vs. curative intent), the receipt of chemotherapy (either adjuvant or neoadjuvant), or the receipt of radiation (either neoadjuvant or adjuvant) were included. With respect to treatment response/cancer outcomes, this review includes all the studies assessing the rate of down-staging, the rate of clinical complete response (cCR) or the pathologic complete response (pCR) following treatment, surgical complications, mortality rate, disease-free survival (DFS), overall survival (OS), or cancer specific survival (CSS). Secondary outcomes included an assessment of the tumor characteristics (e.g., stage at diagnosis, tumor location), the diagnostic work up received, and the broader care preferences and costs of care in rural populations. All the primary and secondary outcomes are briefly summarized in narrative form, with more detail on the primary outcomes provided in tables.

## 3. Results

A total of 118 studies were identified in the initial search, followed by the elimination of 61 studies, which were conducted outside the US and Canada. The remaining 57 articles were retrieved, of which 2 were eliminated for being commentary/editorial in nature. The remaining 55 articles underwent a full-text review, with 30 articles included for the final analysis (Figure 2), 24 from the US and 6 from Canada. Among the included studies, most used large state-wide or nationwide databases, with overlapping patient populations, making it impossible to assess the number of unique patients represented. Notably, there was no consistent definition of rurality across the studies (Table 1).

### 3.1. Treatment Modalities

Twenty-one studies addressed the treatment of rectal cancer in rural patient populations: 14 addressed surgical treatment (Table 2), 7 addressed chemotherapy (Table 3), 13 addressed radiation therapy (Table 4), and 6 addressed multimodal therapy, combining 2+ of the aforementioned modalities (Table 5).

Fourteen studies addressed surgical treatment, including the type of surgery performed, the use of minimally invasive techniques, and the timing of surgery during the course of treatment. Four studies found that a similar proportion of patients underwent surgical resection for rectal cancer in urban versus rural settings and in regard to those who were closer or further from the treatment centers [47,51,55,56]. One study showed that rural patients were less likely to receive surgery than their urban counterparts [57]. In contrast, two studies found that rural patients or those who traveled longer distances for treatment had higher rates of surgical treatment, although the likelihood of undergoing surgery varied according to whether rurality was measured by urban/rural designation vs. distance from the treatment center [34,40]. It is unclear from these studies how the non-surgical patients were managed, such as whether they were lost to follow up or purposefully subject to a watch-and-wait protocol. No studies directly discussed a watch-and-wait treatment strategy in rural populations. Three studies found that rural or long-distance travel patients were less likely to receive minimally invasive surgery or palliative surgery [31,42,46]. However, the studies found no difference in the rate of R0 resection [53] or the rates of sphincter-preserving surgery (e.g., LAR vs. APR; [28,45]).

With regard to timing, one study demonstrated that rural patients, as compared to metro- or micropolitan patients, had a greater likelihood of surgery within 180 days of diagnosis, but longer average wait times until surgery [31]. A second study found similarly mixed evidence on waiting times depending on whether they were evaluated by distance traveled or rural designation, with patients living over 100 km from the treatment center having longer wait times, but not those in designated rural population centers [47]. Overall, rural patients do not consistently experience significant delays to surgery; they undergo surgical resection at similar rates as those patients in urban centers, and, while they undergo similar types of surgery (APR/LAR), it is less often minimally invasive.

The studies that addressed chemotherapy, radiation therapy, and multimodal therapy found fewer differences than the studies addressing surgical treatment. Six of eight studies showed no difference in the likelihood of undergoing chemotherapy [28,39,43,46,47,51] and five of eight studies showed no difference in the likelihood of undergoing radiation therapy for rural patients [47,51,52,55,56]. The differences between rural and urban patients included increased waiting times for treatment [46], increased neoadjuvant chemotherapy use [31], a lower rate of neoadjuvant radiation use despite similar overall rates of radiation therapy [54], and a higher likelihood of receiving under-dosed radiation therapy in rural patients [41]. A separate study showed a significant decrease in radiation utilization in patients living >100 km from the treatment center [53]. In regard to the studies assessing the utilization of multimodal therapy in rural patients, two studies found no difference in the rates of surgery combined with radiation or chemoradiation [47,55], one study found higher utilization of neoadjuvant chemoradiation in rural patients [49], one study found higher utilization of surgery and radiation within 180 days of diagnosis in rural patients [31], and one study found higher utilization of chemoradiation in urban patients compared to those in small rural communities, but not in large rural communities [33].

### 3.2. Oncologic Outcomes/Response to Treatment

Of the 13 studies addressing the response to treatment in rural rectal cancer patients (Table 6), rural patients generally fared neutral to worse than their urban counterparts. Five studies showed significantly worse outcomes for rural patients, including lower rates of pathologic complete response [30], a higher 30-day readmission rate [35], higher recurrence rates [53], and lower survival [39,47]. One of these studies found that rural patients fared worse in terms of recurrence and survival than their urban counterparts and that rural patients treated in urban centers also fared worse than their neighbors treated closer to home [53]. Another study similarly demonstrated that a travel distance greater than 100 km or greater than 1 h from the closest available treatment center was associated with significantly decreased OS and DFS, although this was not evident when analyzed according to the rural vs. urban population center, suggesting that the distance traveled may impact care more than the simple rural–urban classification [39]. This study was of particular importance as it controlled for distance outliers (>300 km) and analyzed the distance traveled compared to the closest appropriate hospital, not the actual treating hospital [39]. These results are supported by a second study showing worse CSS for patients living far from the treatment center, but no difference was seen based on the urban/rural designation [47]. A final study found that despite overall trends of a falling mortality for rectal cancer, the age-adjusted mortality rate for rural patients has been increasing since 1999 [29].

However, four studies found a minimal to no difference in the response to treatment, as measured by the overall and disease-specific survival [28,46,54,55]. One study showed improved outcomes for rural patients compared with urban patients, finding that patients traveling the furthest distance had superior outcomes in terms of their 30-day mortality compared to those traveling the smallest distance [34]. However, this study evaluated the distance to the treatment center (not the closest treatment center) and did not exclude distance outliers, so it may not truly represent the situation in regard to rural patients. A second study demonstrated some survival benefits for rural patients compared to urban patients, including higher survival among rural patients who did not undergo surgery compared to urban patients who did not undergo surgery, although whether the patients declined surgery or were enrolled on watch-and-wait protocols was unclear [50].

### 3.3. Presentation and Diagnostic Work Up

Five studies addressed presentation and diagnostic work up. One study found no significant difference in acuity at presentation, as defined by presentation in the hospital or ED for cancer-related symptoms compared to evaluation in a clinic, between urban and rural patients [32]. Two studies addressed colonoscopies [43,44]; one of which, evaluating only rural rectal cancer patients, found that only ~6% of patients over fifty years old had received a screening colonoscopy [44]. Rural and/or long-distance travel patients were found to have a longer median time between diagnosis and a medical oncology consultation [47] and their pathology reports were significantly less likely to conform to reporting guidelines, particularly regarding the histologic grade, radial margin, and distance from the invasive carcinoma to the closest margin [37].

### 3.4. Tumor Characteristics

Nine studies offered insight into the distribution of the stage at presentation in rural patients. Many of the studies restricted their population to only stage II and III disease; however, one study, examining only rural patients in Appalachia, found that, regardless of the age of onset (early vs. late), there was a roughly similar distribution of the stages, including ~48% of patients diagnosed at an early stage (stage 0-II; [44]). Four studies found no statistically significant difference in the stage at diagnosis between rural and urban/metropolitan patients [28,33,43,53], whereas three studies found that rural patients presented with a lower stage disease compared with their urban counterparts [39,46,47]. Additionally, one study found no difference in the distribution of high, mid, and low rectal cancers between urban and rural groups [33].

### 3.5. Preferences, Costs, and Other Outcomes

Only two studies directly addressed the preferences of rural rectal cancer patients. One found no significant difference in the factors considered by urban vs. rural patients when choosing a hospital/surgeon, such as being directed by a referring provider, a personal relationship, or reputation [36]. A second study found that rural rectal cancer patients treated in urban centers listed factors such as the hospital volume, reputation, or the occurrence of a trusted referral by their referring provider as being major motivators in regard to their decisions on which hospital/surgeon to opt for, with high costs of travel, logistical inconvenience, and difficulties with family support being major drawbacks [48]. Two studies found that rural rectal cancer patients were more likely to be treated in low-volume and/or critical access centers [33,38]. One study found longer drive times for rural patients, particularly for those electing to be treated at high-volume hospitals [43]. Finally, one study found that rural patients had statistically significant increased costs in regard to accessing care compared to those living in urban settings [28].

## 4. Discussion

This is the first scoping review to assess the disease presentation, contemporary management, and outcomes of rural patients with rectal cancer. We found that patients in rural and urban areas present with a similar stage of disease. Additionally, while rural patients generally travel further for care, the receipt of radiation, chemotherapy, and surgical management are similar to urban patients, aside from lower rates of minimally invasive surgery and some suggestion that higher rates of under-dosed radiation occur and that there is less use of neoadjuvant radiotherapy. However, rural patients experience higher costs of care and may have slightly worse oncologic outcomes. Importantly, many gaps in the literature remain.

While it is often noted anecdotally that rural patients present with a higher stage of disease or later in the disease course, the current literature suggests that rural and urban patients present with a similar stage of disease. However, many studies found that rural patients have worse oncologic outcomes, raising questions about factors unique to the care of rural patients, which may impact outcomes. One study highlighted lower neoadjuvant radiotherapy use in rural patients [54] which, given the known benefit of neoadjuvant radiotherapy [14], in coordination with a second study finding higher rates of under-dosed radiotherapy [41], may explain some of these disparate outcomes. Moreover, while the studies generally found similar rates of chemotherapy and radiation between rural and urban patients, few studies specified whether chemoradiation treatments were being administered in the adjuvant or neoadjuvant setting, as neoadjuvant chemoradiation was not the standard of care at the time that many of these studies were conducted [3]. As such, it is difficult to make definitive conclusions about whether rural rectal cancer patients are receiving the standard of care.

With regard to the surgical management of rural patients, aside from lower rates of minimally invasive surgery, the frequency and type of surgery performed is comparable to urban patients. While this is reassuring, the treatment for rectal cancer increasingly emphasizes organ preservation, with the acceptance of the watch-and-wait strategy by the NCCN in 2023 [11]. Currently, no studies discuss the utilization of watch-and-wait approaches in rural settings. As this strategy continues to gain traction in the US, it will be an important aspect to study in rural communities given the unique challenges they face in regard to accessing care.

Quality of life and patient preferences are important and are understudied components to consider when deciding how to optimize care for rural rectal cancer patients. The current literature on this topic is sparse, limiting the conclusions that we can draw here, but tends to indicate that high cost and difficulties in terms of family support are salient variables, even for patients who opt to be treated at regional centers. Additionally, outside of the colorectal literature, one study out of Vermont found that rural patients would accept a substantially increased mortality risk in order to be able to receive treatment locally: 45/100 patients would accept double and 23/100 patients would accept quadruple the mortality risk in order to receive treatment at a local center [58]. Another study from the family medicine literature showed that patients are more likely to trust and comply with physician recommendations if they perceive their physician as being from a similar background to them, with similar belief systems and values [59]. Taken together, the studies above indicate that rural patients may be willing to sacrifice their health in favor of their quality of life, and that they may respond better to being treated by physicians who are members of their local community.

Despite some evidence that rural patients would prefer to be treated in or near their home communities, nationwide trends in rectal cancer treatment favor the regionalization of care given the improvement in patient outcomes. This is increasingly pushing rural patients toward urban centers. Inherent in this framework is the assumption that the cumulative costs, financial, logistical, social, and in regard to morbidity/mortality, will be outweighed by the improved oncologic outcomes. Unfortunately, although the benefits of centralizing care in regard to HVHs and HVSs are clearly demonstrated for the population as a whole, the majority of whom live in urban/metropolitan areas, the same cannot necessarily be said for rural patients. This problem is likely to intensify, as one study found that if rectal cancer treatment were to be further restricted to HVHs only, there would be a 321% increase in the number of patients in New York State that had to travel 50+ miles for surgery [60]. This highlights the potential unintended consequences of regionalizing care for rural patients who are already subject to a disproportionately higher travel burden and associated costs, without reaping the same oncologic benefits as compared to their urban counterparts. Moreover, this raises the possibility that efforts at regionalization will disincentivize rural patients from seeking care, instead of redirecting them to the highest quality of care available.

This review is not without its limitations. Given the use of overlapping databases, the total population of the patients covered by the studies could not be assessed. This combined with the lack of standardization of the definitions meant a meta-analysis was not feasible. Furthermore, the data are limited to the US and Canada, which limits its generalizability, but also raises important differences between the two countries and their healthcare systems. Lastly, studying this population is challenging given the lack of consensus around the definition of “rural” [4,5,6,7]. Canada has its own classification system [27], which is distinct from the two prevailing methods used in the US to define “rural”: the rural–urban continuum codes (RUCC), used to define counties, and the rural–urban commuting areas (RUCA), used to define census tracts [25,26]. Moreover, the thresholds according to which codes are classified as “rural” vs. “urban” vary from study to study, as demonstrated in this article. Due to multiplicative definitions and a lack of generalizability of these metrics, researchers in both countries have developed alternative metrics for measuring rurality, including the distance to the treating facility, the distance from the closest available facility, and population density (Table 1). This has major implications for studying this population, as seen in the differential distribution of rurality across the US and Canada using different definitions alone (Figure 1; [4,5]). Moreover, different definitions complicate the ability to ensure that a similar population is captured across studies, limiting comparisons. The field would certainly benefit from at least some degree of standardization in how we define and quantify rurality, even if that is only achievable on a country-by-country basis.

Given the current system, there is no single, easy solution to rural healthcare access for rectal cancer patients. Significantly more research is needed to fully understand the scope of the problem and the impacts of current healthcare trends, from regionalization to changing guidelines to the expansion of telehealth services. Future studies are needed to evaluate the impacts of contemporary guidelines on the rectal cancer outcomes in rural communities, including more involvement of rural treatment centers in on-going clinical trials. Lastly, there is an important role for more qualitative research here in regard to the preferences of rural patients with rectal cancer, which would help to guide co-decision making. However, any solution will likely require increased investment in rural healthcare facilities, enhanced collaboration between rural community hospitals and larger academic centers, better integrated referral networks, and more focus on the training, recruitment, and retention of rural physicians.

## 5. Conclusions

The aim of this review was to evaluate the contemporary management of rectal cancer in rural populations. Rural patients face unique challenges in regard to care, including increased difficulty in accessing healthcare and a disproportionate increase in age-adjusted rectal cancer mortality compared to urban patients. Consensus around the definition of rurality is necessary to assess this population in further studies in order to bridge the current gaps in the literature, particularly around watch-and-wait approaches in rural populations, the administration of guideline-concordant care, and further exploration into patient preferences in regard to their care. Careful attention to the rural population will be necessary with increasing regionalization of rectal cancer care, including targeted efforts to optimize outcomes in this population, in order to ensure that the needs of this vulnerable population are being adequately considered.

## Figures and Tables

**Table 1:** Included studies, from 2000 to 2024, addressing the care for rural patients with rectal cancer in the United States and Canada.

Study Information	Loc.	Data Source	Primary Outcomes	Measure of Rurality
Peterson K.J. et al. *J. Surg. Res.* 2024 [28]	USA	Single institution retrospective cohort, using EMR chart review	Overall mortality, disease recurrence, and quality of life	Distance to urban/specialized treatment center
Tan J.Y. et al. *Int. J. Cancer* 2024 [29]	USA	US Centers for Disease Control and Prevention’s Wide-Ranging Online Data for Epidemiologic Research	Age-adjusted mortality rate, average annual percent change in colon, rectosigmoid, and rectal cancer	2013 US census classification: urban (large central metro, large fringe metro, medium metro, and small metro counties) and rural (micropolitan non-metro and non-core non-metro counties)
Shulman R.M. et al. *JAMA Netw. Open* 2024 [30]	USA	National Cancer Database	Rate of pCR following neoadjuvant therapy	Assessing urban vs. rural facility, does not specify metrics
Sha S.T. et al. *Adv. Radiat. Oncol* 2023 [31]	USA	Medicare Claims Data	Composite of both proctectomy and RT therapy within 180 d. Subset with proctectomy: pre-op RT, pre-op chemo, and MIS	US census definitions of metropolitan (≥50,000 people), micropolitan (10,000–49,999), or small town/rural (<10,000 people), using patient location not treatment facility location
Sutton T.S. et al. *PLoS One* 2023 [32]	USA	Single institution retrospective cohort at a CoC-accredited tertiary care center, chart review of EMR	Acuity of presentation, stage at diagnosis before and after COVID-19	Rural vs. urban/metropolitan defined as per the Federal Office of Rural Health Policy, assessing patient residence
Goffredo P. et al. *J. Gastrointest. Surg.* 2023 [33]	USA	Iowa Cancer Registry and patient survey	Association of demographics, tumor location, neoadjuvant CRT, sphincter-sparing rates, and hospital volume	Rural–urban commuting area (RUCA): small rural, large rural, urban
Hao S. et al. *Int. J. Colorectal Dis.* 2023 [34]	USA	National Cancer Database	Correlation between distance traveled and receipt of surgery	NCDB uses USDA economic research services definition of rurality: rural (<2500), urban (2500–20,000+ and adjacent to a metro area), and metro (<250,000–1,000,000+ in defined metropolitan areas). Distance traveled to treating hospital measured in quartiles (1st 0–4.6; 2nd 4.6–10.5; 3rd 10.5–25.6; 4th 25.6–4191, all in miles)
Emile S.H. et al. *Surgery* 2023 [35]	USA	Retrospective case-controlled study using NCDB	Rate and predictors of 30-day unplanned readmission and the impact of readmission on short-term mortality and overall survival	Per NCDB user file: metropolitan, namely counties in metro areas with <250,000 to ≥1 million population; urban, namely urban population of 2500 to ≥20,000, adjacent or not adjacent to a metro area; and rural, namely completely rural or <2500 urban population, adjacent or not adjacent to a metro area, itself based on USDA economic research service
Del Vecchio N.J. et al. *Dis. Colon. Rectum* 2022 [36]	USA	Iowa Cancer Registry used to identify patients for survey, as well as for demographic and cancer data	Characteristics of the hospitals where patients choose to receive surgery	Patient zip codes were classified based on 3-tiered rural–urban commuting area scheme (urban, large rural, and small rural)
Sho S. et al. *Dis. Colon. Rectum* 2020 [37]	USA	NSABP R-04 Clinical Trial Data, two reviewers analyzing pathology reports and compared them against College of American Pathologist (CAP) 2013 guidelines	Adherence to the College of American Pathologists guidelines, impact of synoptic reporting, academic status, rural/urban, and hospital size on report quality	American Hospital Association Data Viewer—hospital location data urban/rural
Matthews K.A. et al. *J. Rural Health* 2020 [38]	USA	Iowa Cancer Registry	Travel time for CRC patients measured by time to nearest cancer surgery facility, time to actual cancer surgery facility, and number of bypassed facilities	Rural–urban commuting area (RUCA), categorization B: isolated rural, small rural, large rural, urban
Gotfrit J. et al. *Public Health Pract.* 2020 [39]	Can	CHORD Consortium outcomes database	Impact of distance, drive time, socioeconomic factors on OS and DFS	Distance in hours and km from patient zip code to closest cancer center; 2016 Canadian census database for rural status and population center size
Fields A.C. et al. *J. Surg. Oncol.* 2020 [40]	USA	NCDB	Predictors of surgical refusal	Does not specify what metric they used for classifying a county as urban/rural. Also uses distance tertiles to treatment facility, but they do not specify what distance ranges each of the tertiles represents
Ofshteyn A. et al. *Am. J. Surg.* 2020 [41]	USA	NCDB	Predictors of inadequate radiation dosing	Does not specify what metric they used for “rural” status or how they calculated the distance from the hospital
Springer J.E. et al. *Ann. Surg. Oncol.* 2020 [42]	Can	Retrospective cohort using Canadian Institute of Health Information Discharge Abstract Database (CIHI), Hospital Morbidity Database	Regional rates for laparoscopy use in rectal cancer surgery	Does not specify how they define urban/rural divide. Uses distance from CRS training program
Chioreso C. et al. *Ann. Surg.* 2021 [43]	USA	Retrospective analysis of SEER Medicare Data	Receipt of rectal cancer resection at a HVH and by a HVS/CRS	Rural–urban commuting area classification using zip code from SEER, distance between patient’s zip code and treating hospital
Wolbert T. et al. *Am. Surg.* 2018 [44]	USA	Retrospective study via chart review and Cabell Huntington Hospital Cancer Registry compared to North American Association of Central Cancer Registries and Surveillance, Epidemiology, and End Results Program database	Prevalence of CRC in rural Appalachia	All patients classified as rural based on being from/treated in “Appalachian Tristate Area” of western West Virginia, eastern Kentucky, and southeastern Ohio
Arsoniadis E.G. et al. *Ann. Surg. Oncol.* 2018 [45]	USA	Nationwide Inpatient Sample (NIS) dataset	Compare rate of APR vs. LAR in African American vs. non-African American rectal cancer patients	Assessing location of hospital based on NIS data. Unclear metric/threshold for designation of hospital as urban vs. rural
Lefresne S. et al. *Am. J. Surg.* 2018 [46]	Can	BC Cancer Agency’s (BCCA) Gastrointestinal Cancer Outcomes Unit (GICOU) database	Rates of neoadjuvant RT, chemotherapy, and sphincter-preserving surgery, DFS, OS	Local health authority (LHA) was identified by patient postal code and then determined to be rural (<50% live in community >10,000 people), small (>50% living in community >10,000 people), and large (>95% people living in community >100,000)
Loree J.M. et al. *J. Rural Health* 2017 [47]	Can	BC Cancer Agency’s (BCCA) Gastrointestinal Cancer Outcomes Unit (GICOU) database	Impact of rural/urban and distance on CSS	Canadian Census Analyzer + postal codes: The Canadian census divides “population centers” into small (1000–29,999), medium (30,000–99,999), and large (100,000+). Population centers are areas with at least 1000 people and a population density of at least 400/km^2^. Rural areas are any places outside an indicated population center. They were also evaluated according to whether they traveled >100 km or <100 km to the center at which they received the majority of their care
Nostedt M.C. et al. *Can. J. Surg.* 2014 [48]	Can	Manitoba Cancer Registry Database, survey rectal cancer patient recruited by rectal cancer-treating surgeons, who were identified using a convenience sample	Preferences and factors considered in determining treatment location	Hospitals not in Winnipeg were classified as rural. Surgeons at designated urban and rural hospitals in Manitoba were contacted to refer patients for convenience sample telephone interview
Monson J.T. et al. *Ann. Surg.* 2014 [49]	USA	Retrospective review of NCDB	Adherence to neoadjuvant CRT guidelines in rectal cancer patients by geographic regions, institution volume, and time	Population density of patient residence not otherwise specified
Sankaranarayanan J. et al. *Expert Rev. Pharmacoecon. Outcomes Res.* 2014 [50]	USA	National Cancer Registry	Colon and rectal cancer survival by age, race, rurality, and other socioeconomic factors	OMB metropolitan classification: urban metro (large >1 M or small 50 k–1 M), micropolitan (10 k–50 k), or non-core/non-metro/rural (0–10 k)
Hines R. et al. *Am. J. Public Health* 2014 [51]	USA	Georgia Comprehensive Cancer Registry	Overall survival based on geographic residence/census tract	Rural–urban commuting area (RUCA) primary codes from the US Department of Agriculture: rural (RUCA codes 7–10), suburban (RUCA codes 2–6), and urban (RUCA code 1)
Fleming S.T. et al. *J. Rural Health* 2014 [52]	USA	Cancer Registries of Kentucky, Ohio, Pennsylvania, and North Carolina, Center for Medicare and Medicaid Services Claims database	Guideline concordance in treating rectal cancer measured by adjuvant chemotherapy, ≥12 lymph, and RT	2003 USDA rural–urban continuum codes
Helewa R.M., et al. *Dis. Colon Rectum* 2013 [53]	Can	Manitoba Cancer Registry, local recurrence information from chart review (paper and electronic charts)	Population-based rates and risk factors for recurrence in Manitoba	Patient and hospital performing the surgery were classified as either urban/Winnipeg or rural/Manitoba. Classified by distance traveled from place of residence to CancerCare Manitoba (site of only radiation therapy): <21 km, 21–100 km, 101–500 km, and >500 km
Stewart D.B. et al. *Ann. Surg. Oncol.* 2013 [54]	USA	Pennsylvania Cancer Registry, linked to medical claims data provided by Highmark Inc. (insurance company)	Use of neoadjuvant RT and CSS by hospital type (urban/rural, large/small, academic/community)	Assessing hospital rurality. Rural–urban commuting area codes were mapped to the zip code of the treating hospital
Hines R.B. et al. *J. Rural Health* 2012 [55]	USA	SEER data from participating counties in Georgia (Atlanta Registry and Rural Georgia Registry)	Rate of late stage (III and IV) at diagnosis, treatment received, cancer-specific mortality by geographic area and race	Used the rural–urban continuum code (RUCA) classification: urban counties were those with RUCA codes ≤3, and rural counties were defined as those with RUCA codes ≥6
Sankaranarayanan J. et al. *Am. J. Manag Care* 2010 [56]	USA	Nebraska Cancer Center Registry	Receipt of surgery, radiation, and/or chemotherapy for CRC by anatomic site, residence county (rural/urban), and age	Office of Management and Budget (OMB) classifications into three categories: “urban metro” counties with large (>1 million) or small-metro (50,000 to <1 million residents) central counties; micropolitan counties (centered on urban clusters with 10,000–49,999 residents, plus surrounding counties); and “rural” counties containing a town of 1 to 9999 residents
Esnaola N.F. et al. *Ann. Surg.* 2009 [57]	USA	South Carolina Central Cancer Registry	Odds of surgical resection for rectal cancer by race	Urban/rural classification based on metropolitan statistical area of the county in which the patient resided, as per HHS Office of Rural Health Policy

**Table 2:** Surgical treatment for rural patients with rectal cancer.

Study	Rurality Measure	Surgical Treatment
Peterson K.J. et al. *J. Surg. Res.* 2024 [28]	Distance to treatment center	No statistically significant difference according to long vs. short distance traveled to treatment center in the percentage of patients who underwent LAR, APR, vs. total proctocolectomy (*p* = 0.325).
Sha S.T. et al. *Adv. Radiat.* *Oncol.* 2023 [31]	US census classifications	Rural patients had a statistically significant greater likelihood of proctectomy within 180 d of diagnosis (small/rural 33.2%, micropolitan 28.4%, metropolitan 28.6%; *p* < 0.01). Metropolitan patients had statistically significant fewer days until surgery (80.7 d vs. 87.0 d, *p* = 0.05), a higher percent of patients receiving MIS surgery (61.6% vs. 53.8%, *p* < 0.01), and were more likely to be treated by a colorectal surgeon or surgical oncologist as opposed to a general surgeon (56.1% vs. 44.1%, *p* < 0.01).
Hao S. et al. *Int. J. Colorectal Dis.* 2023 [34]	USDA economic research service classification Distance from treating facility (in quartiles)	There was no statistically significant difference in the receipt of surgery by rural/urban/metro status: metro (ref), urban (0.92, *p* = 0.08), rural (1.02, *p* = 0.89). There was a statistically significant difference in the receipt of surgery between 1st and 4th quartile of the distance traveled to the care provider (with more people in the 4th quartile receiving surgery), but not the 2nd and 3rd: 1st (ref), 4th (1.37, *p* < 0.001).
Fields A.C. et al. *J. Surg. Oncol.* 2020 [40]	Unclear metric for urban/rural Distance to treatment facility (in tertiles)	There was a statistically significant difference in regard to whether patients with stage I rectal adenocarcinoma chose to refuse surgery based on the distance from the facility in the bivariate analysis (1st OR ref; 2nd OR 0.6, *p* < 0.001; 3rd OR 0.3, *p* < 0.001) and multivariate analysis (1st OR ref; 2nd OR 0.6, *p* < 0.001; 3rd OR 0.2, *p* < 0.001). Analysis was not provided for urban–rural designation. There was no statistically significant difference in regard to whether the patients with stage II and III rectal adenocarcinoma chose to refuse surgery based on urban–rural designation in the bivariate analysis (metro OR ref; rural OR 1.0, *p* = 1), but there was in the multivariate analysis (metro OR ref; rural OR 1.5, *p* = 0.02). There was a statistically significant difference in regard to whether the patients with stage II and III rectal adenocarcinoma chose to refuse surgery based on the distance from the facility in the bivariate analysis (1st OR ref; 2nd OR 0.6, *p* < 0.001; 3rd OR 0.5, *p* < 0.001) and multivariate analysis (1st OR ref; 2nd OR 0.7, *p* < 0.001; 3rd OR 0.6, *p* < 0.001).
Springer J.E. *Ann. Surg.* *Oncol.* 2020 [42]	Unclear metric for urban/rural Distance from CRS training program	Patients living >100 km from the colorectal training program were less likely to receive laparoscopic surgery for their rectal cancer than those <25 km (OR 2.38, *p* < 0.001) or those living 26–100 km (OR 1.79, *p* < 0.001) from the program. Rural patients had no statistically significant difference in regard to the receipt of laparoscopic surgery than those living >100 km from the CR fellowship program (OR 0.95, *p* = 0.26). Rural patients were not directly compared to urban patients. A total of 94.2% of patients who received laparoscopic surgery for rectal cancer lived within 100 km of the CR fellowship training facility. A total of 89.9% of patients in low-laparoscopy clusters lived >100 km from a CR fellowship training facility.
Wolbert T. et al. *Am. Surg.* 2018 [44]	All patients designated rural based on being from “Appalachian Tristate Area”	Age <50 years: 13% received APR, 38% received LAR, 13% received polypectomy, and 4% received polypectomy with curative intent. It was not stated what, if any, surgical treatment the remaining patients received. Age >50 years: 13% received APR and 13% received polypectomy. It was not stated what, if any, surgical treatment the remaining patients received.
Arsoniadis E.G. et al. *Ann. Surg. Oncol.* 2018 [45]	Unclear metrics, designated by location of treating hospital	There was no statistically significant difference in the odds of sphincter-preserving surgery for patients treated in rural vs. urban hospitals as a result of multivariate modeling (urban OR ref; rural OR 0.91, 95%CI 0.81–1.02)
Lefresne S. et al. *Am. J. Surg.* 2018 [46]	Local health authority (LHA) size: rural, small, and large	Patients in large urban areas had a slightly higher rate of palliative surgery than small or rural community patients (large LHA 8.7%, small LHA 6.6%, rural LHA 4.4%; *p* = 0.04), but there was no statistically significant difference in regard to the choice of surgical procedures
Loree J.M. et al. *J. Rural Health* 2017 [47]	Canadian population centers according to census: rural, small, medium, and large	No statistically significant difference in the percentage of patients receiving surgery (*p* = 0.73) when evaluated by distance (>100 km vs. <100 km) from the treatment center, which persisted in the multivariate analysis (>100 km OR 0.69, <100 km ref; *p* = 0.34). By population center, a statistically significant difference in the percentage of patients receiving surgery occurred (rural 95.1%, small 92.2%, medium 91.5%, large 92.5%; *p* = 0.02) that did not hold up in the multivariate analysis (rural OR 2.16, *p* = 0.051; large ref). No statistically significant difference in the TME occurred according to distance or population center (*p* = 0.78, *p* = 0.33). The number of days from diagnosis until surgery was longer for those living >100 km from the primary treatment center (61 days vs. 56 days, *p* = 0.013); however, there was no statistically significant difference in the number of days waited until surgery when analyzed according to the population center (*p* = 0.12)
Hines R. et al. *Am. J. Public Health* 2014 [51]	RUCA	No statistically significant differences in regard to the odds of receiving surgery for rectal cancer between urban, suburban, and rural patients
Helewa R.M. et al. *Dis. Colon Rectum * 2013 [53]	Winnipeg vs. non-Winnipeg Manitoba Distance from treating hospital	There were no statistically significant differences in the percentage of patients who achieved R0 resection (Winnipeg–Winnipeg 86.2%, Rural–Winnipeg 88.1%, Rural–Rural 84.1%, *p* = 0.77). There were no statistically significant differences in regard to the type of surgical resection between the residence–hospital location interaction (*p* = 0.26)
Hines R.B. et al. *J. Rural Health* 2012 [55]	RUCA codes	There was no statistically significant difference in regard to the receipt of surgery (OR 0.92, 95%CI 0.51–1.66) comparing rural and urban patients
Sankaranarayanan J. et al. *Am. J. Manag. Care* 2010 [56]	OMB’s classifications of urban metro, micropolitan, and rural	A total of 79.7% of rural patients underwent surgery as opposed to 78% of urban and 77.1% micropolitan patients, which was not statistically significant. The multivariate analysis showed the odds of rural (ref) patients receiving surgery were not statistically significant (*p* = 0.08) from that of urban patients (OR 0.73) and micropolitan patients (OR 0.58)
Esnaola N.F. et al. *Ann. Surg.* 2009 [57]	HHS office of Rural Health Policy’s classification of Metropolitan Statistical Area	There was statistically significant decreased odds of rural patients receiving surgery for rectal cancer (OR 0.59, *p* < 0.001) when compared to urban patients (ref)

**Table 3:** Chemotherapy for rural patients with rectal cancer.

Study	Rurality Measure	Chemotherapy
Peterson K.J. et al. *J. Surg. Res.* 2024 [28]	Distance to treatment center	No statistically significant difference was observed in regard to the percentage of patients who received neoadjuvant chemotherapy (short distance 39%, long distance 51%; *p* = 0.233)
Sha S.T. et al. *Adv. Radiat.* *Oncol.* 2023 [31]	US census classifications	There was no statistically significant difference in the percentage of patients who received chemotherapy within 180 days of diagnosis (rural 38.7%, metro 35.9%, *p* = 0.07), but there was a statistically significant increased neoadjuvant chemotherapy percentage usage in regard to rural patients (44.4% vs. 37.7%, *p* = 0.01)
Gotfrit J. et al. *Public Health Pract.* 2020 [39]	Canadian population centers by census: rural, small, medium, and large Distance in kilometers and hours to closest cancer center	There was no statistically significant difference in regard to the receipt of neoadjuvant chemotherapy for those living >100 km from the treatment center (*p* = 0.44) or for those living >1 h from the treatment center (*p* = 0.36)
Chioreso C. et al. *Ann. Surg.* 2021 [43]	RUCA codes	No statistically significant differences were observed in regard to the medical oncology visits between rural and urban patients. The study does not report directly on the use of chemotherapy
Lefresne S. et al. *Am. J. Surg.* 2018 [46]	Local health authority (LHA) size: rural, small, and large	Median waiting time from diagnosis to cancer center visit was longer for rural LHA than for small LHA and large LHA communities (rural LHA 39 d, small LHA 35 d, large LHA 32 d days; *p* < 0.01). The receipt of chemotherapy was not associated with the patients’ urban/rural status (rural LHA 57%, small LHA 56%, and large LHA 57%; *p* = 0.89)
Loree J.M., et al. J. Rural Health 2017 [47]	Canadian population centers according to census: rural, small, medium, and large	No statistically significant differences were observed in regard to the percentage of patients who received adjuvant chemotherapy when analyzed according to the distance from the primary treatment center (*p* = 0.129), with similar odds between the two groups (<100 km OR ref; >100 km OR 0.90, *p* = 0.52), in the multivariate analysis. A statistically significant difference was observed in regard to the percentage of patients who received adjuvant chemotherapy when analyzed according to the population center (rural 51.6%, small 44.8%, medium 45.8%, large 54.9%; *p* = 0.0005); however, in the multivariate analysis, the odds of receiving chemotherapy were not statistically significantly different between the groups (rural OR 0.8, *p* = 0.15; small OR 0.7, *p* = 0.15; medium OR 0.74, *p* = 0.15; large ref)
Hines R. et al. *Am. J. Public Health* 2014 [51]	RUCA codes	No statistically significant differences were observed in regard to the odds of receiving chemotherapy between urban, suburban, and rural patients

**Table 4:** Radiation treatment for rural patients with rectal cancer.

Study	Rurality Measure	Radiation
Peterson K.J. et al. *J. Surg. Res.* 2024 [28]	Distance to treatment center	The data were just shy of statistical significance in regard to the percentage of patients receiving neoadjuvant radiation according to the distance from the treating facility (long 77%, short 59%, *p* = 0.057)
Sha S.T. et al. *Adv. Radiat.* *Oncol.* 2023 [31]	US census classifications	There were no statistically significant differences in regard to the receipt of radiation within 180 d of diagnosis according to region. In the univariate analysis, a greater percentage of rural patients received neoadjuvant radiation (49.9% vs. 44.4%, *p* = 0.04) compared to metropolitan patients; however, when analyzed further, the adjusted odds ratio was not statistically significant (rural neoadjuvant OR 1.21, metropolitan ref; *p* = 0.1). IMRT was favored in metropolitan areas compared to rural areas (metro 50.6%, rural 38.9%, *p* < 0.01)
Gotfrit J. et al. *Public Health Pract.* 2020 [39]	Canadian population centers according to census: rural, small, medium, and large Distance in hours and kilometers to the closest cancer center	There were also no statistically significant differences in regard to the receipt of neoadjuvant radiation therapy for those living >100 km from the treatment center (*p* = 0.91) or for those living >1 h from the treatment center (*p* = 0.23)
Ofshteyn A. et al. *Am. J. Surg.* 2020 [41]	Unclear metric for urban/rural Distance from hospital	Rural patients were more likely than urban or metropolitan patients to receive inadequate radiation dosing (rural 7.5%, urban 6.5%, metro 5.1%; *p* < 0.001), as were patients who had to travel a longer distance to the hospital (<50 mi 5–5.6%, >50 mi 6.2–6.8%; *p* = 0.034), as shown by the univariate analysis. The increased odds of inadequate radiation dosing for rural patients compared to metropolitan patients remained statistically significant in the multivariate analysis (metro OR ref; rural OR 1.42, *p* = 0.035)
Chioreso C. et al. *Ann. Surg.* 2021 [43]	RUCA codes	No statistically significant differences in regard to the radiation oncology visits between rural and urban patients. The study does not report directly on the use of radiation therapy
Lefresne S., et al. *Am. J. Surg.* 2018 [46]	Local health authority (LHA) size: rural, small, and large	The median waiting time for RT was 54 days and did not vary according to the urban/rural LHA (*p* = 0.41). There were no differences according to the urban/rural LHA in regard to whether a patient received any RT (*p* = 0.8), short vs. long course fractionation RT regimen (*p* = 0.42), or pre-op vs. post-op RT timing (*p* = 0.74)
Loree J.M. et al. *J. Rural Health* 2017 [47]	Canadian population centers according to census: rural, small, medium, and large	There were no statistically significant differences in regard to the percentage of patients receiving radiation therapy when compared according to the distance from the primary treatment center (*p* = 0.097), with a similarly non-statistically significant difference in odds (<100 km ref; >100 km OR 1.28, *p* = 0.3), in the multivariate analysis. A statistically significant difference in regard to the percentage of patients receiving radiation therapy was observed when analyzed according to the population center (rural 83.5%, small 88.1%, medium 87.8%, large 88.0%, *p* = 0.0057); however, the decreased odds of receiving radiation therapy if the patient was in a rural setting as compared to a large population center was not statistically significant (large OR ref; rural OR 0.68, *p* = 0.08) in the multivariate analysis
Hines R. et al. *Am. J. Public Health* 2014 [51]	RUCA	No statistically significant differences were observed in regard to the odds of receiving radiation therapy between urban, suburban, and rural patients
Fleming S.T. et al. *J. Rural Health* 2014 [52]	RUCC	No statistically significant differences were observed in regard to the receipt of radiation therapy for metropolitan (58.3%) vs. non-metropolitan (53.4%) stage III rectal cancer patients living in Appalachia
Helewa R.M. et al. *Dis. Colon Rectum* 2013 [53]	Winnipeg vs. non-Winnipeg Manitoba Distance from treating hospitals	There was a statistically significant difference in regard to the receipt of radiation therapy when assessed according to the distance traveled to the radiation center, driven by the significantly decreased odds of receiving radiation therapy if living 101–500 km away from CancerCare Manitoba (<21 km OR ref; 21–100 km OR 0.76, 95%CI 0.34–1.72; 101–500 km OR 0.23, 0.08–0.63; >500 km OR 0.8, 0.10–6.36; *p* = 0.032)
Stewart D.B. et al. *Ann. Surg. Oncol.* 2013 [54]	RUCA (by hospital location)	In the univariate analysis, there was no statistically significant difference in the use of radiation therapy in urban vs. rural hospitals treating patients with stage II and III rectal cancer (rural 57.7%, urban 62.2%; *p* = 0.66); however, there was a statistically significant increase in use of neoadjuvant radiation in urban vs. rural hospitals (rural 19.2%, urban 39.2%; *p* = 0.046)
Hines R.B. et al. *J. Rural Health* 2012 [55]	RUCA codes	There was no statistically significant difference in regard to the receipt of radiation (OR 0.70, 95%CI 0.43–1.15) when comparing rural and urban patients
Sankaranarayanan J. et al. *Am. J. Manag. Care* 2010 [56]	OMB’s classification of urban metro, micropolitan, and rural	A total of 37.7% of rural patients received radiation therapy as opposed to 43.5% urban and 42.4% micropolitan patients, which was not statistically significant. The multivariate analysis showed odds of rural rectal cancer patients (ref) receiving radiation to be not statistically significantly different (*p* = 0.32) from urban patients (OR 1.27) and micropolitan patients (OR 1.31)

**Table 5:** Multimodal treatment (CT, RT, and/or surgery) in rural patients with rectal cancer.

Study	Rurality Measure	Combined Therapy
Sha S.T. et al. *Adv. Radiat.* *Oncol.* 2023 [31]	US census classifications	The combined outcome of both surgery and radiation within 180 d of diagnosis showed that there was a statistically significant higher percentage of rural patients falling into this category (rural 17.8%, metro 13.7%, *p* < 0.01); likewise, on further analysis, the adjusted sub-hazard ratio in regard to the receipt of both radiation and surgery in <180 days favored rural patients (rural ASHR 1.15, metropolitan ref; *p* = 0.05)
Goffredo P. et al. *J. Gastrointest.* *Surg.* 2023 [33]	RUCA codes	There were statistically significantly decreased odds of receiving chemoradiation when comparing small rural (OR 0.49, *p* = 0.01) to urban (ref) patient population. There was no statistically significant difference in regard to the likelihood of treatment with chemoradiation between large rural and urban populations
Loree J.M. et al. *J. Rural Health* 2017 [47]	Canadian population centers according to census: rural, small, medium, and large	The odds of receiving surgery and radiation were not statistically significantly different according to the distance from the primary treatment center (<100 km ref; >100 km OR 1.1, *p* = 0.67) or according to the population center (rural OR 0.9, *p* = 0.56; small OR 0.88, *p* = 0.7; medium OR 1.1, *p* = 0.73; large OR ref) in the multivariate analysis. The odds of receiving surgery, chemotherapy, and radiation were not statistically significantly different according to the distance from the primary treatment center (<100 km OR ref; >100 km OR 0.84, *p* = 0.31) or according to the population center (rural OR 0.83, *p* = 0.22; small OR 0.65, *p* = 0.09; medium OR 0.75, *p* = 0.16; large ref) in the multivariate analysis
Monson, J.T. et al. *Ann. Surg.* 2014 [49]	Population density and patient’s residence not otherwise specified	Patients in rural counties were more likely to receive neoadjuvant chemoradiation than those in metropolitan counties (OR 1.36, *p* < 0.001) in the multivariate analysis
Hines R.B. et al. *J. Rural Health* 2012 [55]	RUCA codes	There was no statistically significant difference in regard to the receipt of combined surgery and radiation (OR 0.78, 95%CI 0.46–1.33) when comparing rural and urban patients

**Table 6:** Outcomes/response to treatment in rural rectal cancer patients.

Study	Rurality Measure	Outcomes/Response to Treatment
Peterson K.J. et al. *J. Surg. Res.* 2024 [28]	Distance to urban/specialized treatment center	According to the distance traveled, no statistically significant differences were observed in regard to the 2-year mortality, 5-year mortality, overall mortality, or recurrence
Gotfrit J. et al. *Public Health Pract.* 2020 [39]	Distance in hours and km to closest cancer center; 2016 Canadian census database urban/rural classification	A distance >100 km (HR 1.59 *p* = 0.006) and a driving time >1 h (HR 1.57, *p* = 0.003) both decreased the OS in the univariate analysis. Only a driving time >1 h was significant in the multivariate analysis (HR 1.6, *p* = 0.002). The same pattern was seen in regard to the DFS, with a distance >100 km (HR 1.16 *p* = 0.01) and drive time >1 h (HR 1.5, *p* = 0.002), in the univariate analysis, but only the drive time >1 h (HR 1.47 *p* = 0.0003) in the multivariate analysis. Designated rural vs. urban areas did not affect the OS or DFS. Recurrence (both local and distant) was higher in patients living >100 km (*p* = 0.02) and >1 h (*p* = 0.007) from the cancer center (31%) compared to those living closer (22%, *p* = 0.02)
Lefresne S. et al. *Am. J. Surg.* 2018 [46]	Patients’ local health authority (LHA) was identified and classified as rural/small/large, based on population size	No difference was observed in the median time to follow up according to the urban/rural LHA (33 months). No statistically significant difference between urban/rural LHA disease recurrence (*p* = 0.99), locoregional recurrence (*p* = 0.88), or development of distance metastasis (*p* = 0.87). At 5 years, in regard to the Kaplan–Meier analysis, the DFS (rural LHA 84%, small LHA 85%, large LHA 86%; *p* = 0.98) and OS (rural LHA 58%, small LHA 59%, large LHA 57%; *p* = 0.99) were not different. This held true in the multivariate analysis of the DFS (rural LHA HR 0.95, small LHA HR 0.97, large LHA HR ref) and OS (rural LHA HR 1.01, small LHA HR 1.02, large LHA HR ref)
Loree J.M. et al. *J. Rural Health* 2017 [47]	Canadian census population center classification of small, medium, large, and rural	The Kaplan–Meier curves for CSS and OS were not different when assessed according to the population center (CSS *p* = 0.18, OS *p* = 0.36) or according to the distance (CSS *p* = 0.88, OS *p* = 0.47). In the multivariate analysis, CSS was statistically significantly decreased in those living >100 km from their primary treatment center (HR 1.39, *p* = 0.031) and increased in those living in small population centers (HR 0.42, *p* = 0.001). No differences in the CSS were observed between large, medium, and rural communities in the multivariate analysis. No differences in the OS in the multivariate analysis were observed when analyzed according to the distance traveled (*p* = 0.16). A statistically significant increase in the OS for those living in small population centers (HR 0.58, *p* = 0.011) was observed in the multivariate analysis, but not in regard to large, medium, and rural communities
Helewa R.M. et al. *Dis. Colon Rectum* 2013 [53]	Winnipeg vs. non-Winnipeg Manitoba Distance from treating hospitals	The unadjusted rectal cancer local recurrence rates were higher for rural patients (2 yr 12.6%, 3 yr 16.2%, 5 yr 27.5%) when compared with Winnipeg patients (2 yr 5.3%, 3 yr 8.3%, 5 yr 10.3%; *p* = 0.0003). All the rural patients had higher recurrence rates than their urban counterparts: Winnipeg–Winnipeg treated (2 yr 5.3%, 3 yr 8.4%, 5 yr 10.3%), rural–Winnipeg treated (2 yr 14.3%, 3 yr 14.3%, 5 yr 28.7%), and rural–rural treated (2 yr 11.5%, 3 yr 17.7%, 5 yr 27.2%; *p* = 0.0013). In the regression analysis, rural residents had a higher local recurrence rate (rural–Winnipeg surgery HR 3.47, rural–rural surgery HR 2.98, Winnipeg–Winnipeg surgery ref; *p* = 0.0003). Lastly, the OS was decreased in the rural patients (HR 1.90, *p* = 0.003) compared to Winnipeg patients (ref)
Hao S. et al. *Int. J. Colorectal Dis.* 2023 [34]	NCDB uses USDA economic research classification: rural, urban and metro Distance traveled to treating facility in quartiles	Statistically significant differences were observed in the 30 d mortality according to the quartile of the distance traveled (1st 1.6%, 2nd 1.3%, 3rd 1%, 4th 0.8%; *p* < 0.001) and the odds of 30 d mortality (1st OR ref; 2nd OR 0.8, *p* = 0.05; 3rd OR 0.59, *p* < 0.01; 4th OR 0.47, *p* < 0.01)
Emile S.H. et al. *Surgery *2023 [35]	USDA economic research service classification: metropolitan, urban, and rural	The multivariate regression showed that rural patients had a higher rate of 30 d readmission than metropolitan patients (OR 1.65, *p* = 0.0004)
Wolbert T. et al. *Am. Surg.* 2018 [44]	All patients designated as rural based on being from “Appalachian Tristate Area”	No difference was observed in the OS between rural patients with early vs. average onset rectal cancer (younger 75%, older 60.2%; *p* = 0.128)
Sankaranarayanan J. et al. *Expert Rev. Pharmacoecon. Outcomes Res.* 2014 [50]	OMB metropolitan classification: urban metro, micropolitan, or non-core/non-metro/rural	No differences were observed in the survival time in months (*p* = 0.29) for rural, urban, and micropolitan patients. In the multivariate regression analysis, urban patients had statistically significantly decreased survival compared with rural patients (rural ref; HR 1.25, *p* = 0.049) when controlling only for demographic factors. However, when controlling for other factors, including the stage, treatment, and other interaction variables, there was no difference in urban/rural survival. When further broken down into the patients who did not undergo surgery, there was a statistically significantly lower survival rate for micropolitan (HR 2.01, *p* = 0.0005) and urban (HR 1.49, *p* = 0.03) patients when compared with rural (ref) patients. No difference in survival according to the urban/rural status was observed when patients underwent surgery
Stewart D.B. et al. *Ann. Surg. Oncol.* 2013 [54]	RUCA codes (treating hospital)	No difference in the CSS curve was observed for rectal cancer patients treated in rural vs. urban hospitals (*p* = 0.83). In the multivariate analysis, there was no difference in the rate of death from rectal cancer in regard to urban vs. rural hospitals (rural HR ref; urban HR 0.954, *p* = 0.9)
Hines R.B. et al. *J. Rural Health* 2012 [55]	Unclear metric, mentions both RUCC and RUCA	No difference in rectal cancer-related death was observed between urban (ref) and rural rectal cancer patients, both overall and when broken down by stage: overall (HR 1.13, 95%CI 0.91–1.41), in situ and stage I (HR 1.29, 95%CI 0.92–1.81), stage II and III (HR 1.17, 95%CI 0.92–1.50), stage IV (HR 1.09, 95%CI 0.73–1.61)
Tan J.Y. et al. *Int. J. Cancer* 2024 [29]	US census classification: urban and rural	The age-adjusted mortality rate (per 100,000) increased from 1999 to 2020 (from 2.95 to 3.01) for rural rectal cancer patients, while decreasing for urban patients (from 3.03 to 2.38); APC for urban patients −1.21 (95%CI −1.48 to −0.95) compared with rural patients, APC +0.10 (95%CI −0.24 to 0.44)
Shulman R.M. et al. *JAMA Netw. Open* 2024 [30]	Assessing urban vs. rural facility, metrics unspecified	According to the urban/rural treatment facility, a statistically significant difference in the odds of a complete pathologic response in rural facilities (0.8) was observed compared to large metro (ref) facilities. No difference was observed in regard to tumor down-staging

## Abbreviations

The following abbreviations are used in this manuscript:

CRS	Colorectal surgery
cCR	Clinical complete response
pCR	Pathologic complete response
HVH	High-volume hospital
HVS	High-volume surgeon
RUCC	Rural–urban continuum codes
RUCA	Rural–urban commuting areas
DFS	Disease-free survival
OS	Overall survival
CSS	Cancer-specific survival
LAR	Low anterior resection
APR	Abdominoperineal resection
LHA	Local health authority
OR	Odds ratio
HR	Hazard ratio
APC	Annual percent change
CI	Confidence interval

## Appendix A

**Table A1:** Inclusion criteria for all studies included in this review.

Study Information	Loc.	Data Source	Inclusion Criteria
Peterson K.J. et al. *J. Surg. Res.* 2024 [28]	USA	Single institution retrospective cohort using EMR chart review	Included: inpatient, >18 yrs, rectal, underwent proctectomy between 1 January 2014 and 1 January 2018, residing within WI or WI-bordering state Excluded: outside institution surgery, trans-anal excision, multi-visceral excision, metastatic at presentation, had histologic diagnosis other than rectal adenocarcinoma
Tan J.Y. et al. *Int. J. Cancer* 2024 [29]	USA	US Centers for Disease Control and Prevention’s Wide-Ranging Online Data for Epidemiologic Research (CDC WONDER) from 1999 to 2020	CRC (ICD-10 codes [ICD-10] C18.0–C18.9 and C19–C20) as the underlying cause of death
Shulman R.M. et al. *JAMA Netw. Open* 2024 [30]	USA	National Cancer Database	Patients with locally advanced rectal cancer treated with neoadjuvant therapy and surgery between 1 January 2004 and 31 December 2017
Sha S.T. et al. *Adv. Radiat. Oncol.* 2023 [31]	USA	Medicare Claims Data	Had fee-for service Medicare Claims, 1 April 2016 to 30 September 2018, non-metastatic rectal cancer Excluded: age <65, ESRD, stage IV disease, had pre-existing cancer in look-back period 1 October 2015 to 31 March 2016, nursing home residents, underwent trans-anal excision
Sutton T.S. et al. *PLoS One* 2023 [32]	USA	Single institution retrospective cohort at a CoC-accredited tertiary care center, chart review of EMR	Patients >18 yrs, rectal adenocarcinoma, 1 January 2016 to 12 December 2021, tumors of upper/middle/lower rectum as confirmed by endoscopy, as measured 15 cm from anal verge OR receiving therapy consistent with rectal adenocarcinoma (e.g., RT)
Goffredo P. et al. *J. Gastrointest. Surg*. 2023 [33]	USA	Iowa Cancer Registry and Patient survey	Inclusion: adults with microscopically confirmed stage II and III rectal cancer between 2013 and 2017 who received cancer-directed surgery with curative intent, alive as of October 2018
Hao S. et al. *Int. J. Colorectal Dis.* 2023 [34]	USA	National Cancer Database (NCDB)	Inclusion: patients with primary rectal adenocarcinoma, between 2004 and 2017, only stage II and III Exclusion: rectal cancer not primary/no rectal cancer, clinical stage missing, stage 0/I/IV, treated at multiple facilities, first treatment unknown
Emile S.H. et al. *Surgery* 2023 [35]	USA	Retrospective case-controlled study using NCDB	Inclusion: non-metastatic rectal adenocarcinoma, underwent proctectomy, between 2010 and 2017 Exclusion: metastatic disease, unknown stage, no surgery, local excision only, total proctocolectomy, unknown surgery type, patients who had planned readmission, or where information on 30 d readmission was missing
Del Vecchio N.J. et al. *Dis. Colon Rectum* 2022 [36]	USA	Iowa Cancer Registry to identify patients for survey, as well as for demographic and cancer data	Iowa residents, age > 18, microscopically confirmed stage II and III rectal cancer, between 2013 and 2017, who received cancer-directed surgery, alive as of October 2018, cognitively/physically able to participate in survey
Sho S. et al. *Dis. Colon Rectum* 2020 [37]	USA	NSABP R-04 Clinical Trial Data, two independent reviewers reviewed pathology reports from study patients between 2004 and 2010 and compared those reports against College of American Pathologist (CAP) 2013 guidelines	Rectal adenocarcinoma, resection July 2004–August 2010
Matthews K.A. et al. *J. Rural Health* 2020 [38]	USA	Iowa Cancer Registry	Iowa residents; diagnosed with rectal cancer, 2010–2014, received surgery, histologies, mucinous adenocarcinoma, signet ring cell carcinoma Exclusion: no surgery, two or more tumors diagnosed within study period, unstaged tumors, select histologies, missing treatment facility information
Gotfrit J. et al. *Public Health Pract.* 2020 [39]	Canada	CHORD Consortium outcomes database	Inclusion: patients from BC, Alberta, Ontario, and Newfoundland, 2005–2013, locally advanced rectal cancer (stage II and III), underwent curative intent neoadjuvant chemoradiation followed by surgery Exclusion: prior malignancy (except non-melanoma skin cancer)
Fields A.C. et al. *J. Surg. Oncol.* 2020 [40]	USA	NCDB	Inclusion: patients with rectal cancer, stage I–III, age >18, with survival data available, either underwent surgery with curative intent OR refused surgery Exclusion: rectosigmoid cancer, stage IV, local excision, ineligible for surgery
Ofshteyn A. et al. *Am. J. Surg.* 2020 [41]	USA	NCDB	Inclusion: age 18+, between 2006 and 2014, clinical stage II and III rectal cancer, received neoadjuvant radiation prior to curative proctectomy Exclusion: patients receiving >5040 cGy radiation, patients who received intentional short-course radiation (2500 cGy or 2000–3000 cGy in 5–10 fractions)
Springer J.E. et al. *Ann. Surg. Oncol.* 2020 [42]	Can	Retrospective cohort using Canadian Institute of Health Information Discharge Abstract Database (CIHI), Hospital Morbidity Database	Inclusion: age 18+, underwent elective rectal resection for rectal cancer, April 2008–March 2014, in Canada Exclusions: Quebec
Chioreso C. et al. *Ann. Surg.* 2021 [43]	USA	Retrospective analysis of SEER Medicare Data	Inclusion: stage II and III primary rectal adenocarcinoma, between 2007 and 2011, not diagnosed at time of death/autopsy Exclusion: prior cancer within 6 m of diagnosis, died within 3 m of diagnosis, patients whose zip code did not have a corresponding RUCA classification
Wolbert T. et al. *Am. Surg.* 2018 [44]	USA	Retrospective chart review and Cabell Huntington Hospital Cancer Registry vs. North American Association of Central Cancer Registries and SEER data	Rectal cancer patients with lower margin <15 cm from anal verge, between 2003 and 2016, living in the Appalachian Tristate Area
Arsoniadis E.G. et al. *Ann. Surg. Oncol.* 2018 [45]	USA	Nationwide Inpatient Sample (NIS) dataset	Inclusion: patients with ICD-9 codes for rectal cancer, 1998–2012, who received proctectomy (LAR ± colostomy or APR) Exclusion: patients with concurrent excluding diagnoses (carcinoids, inflammatory bowel disease, regional enteritis, rectosigmoid cancer), metastatic disease, surgery that involved resection of other pelvic organ, trans-sacral surgery
Lefresne S. et al. *Am. J. Surg.* 2018 [46]	Can	BC Cancer Agency’s (BCCA) Gastrointestinal Cancer Outcomes Unit (GICOU) database	Inclusion: patients with stage II and III rectal adenocarcinoma, between 2004 and 2009
Loree J.M. et al. *J. Rural Health* 2017 [47]	Can	BC Cancer Agency’s (BCCA) Gastrointestinal Cancer Outcomes Unit (GICOU) database	Inclusion: patients diagnosed with stage II and III rectal cancer, between 1 January 1999 and 31 December 2009, referred to BCCA center Exclusion: primary colon cancer, synchronous colon cancer, histology other than rectal adenocarcinoma, timing of radiation therapy not assessed in patients diagnosed before 2005
Nostedt M.C. et al. *Can. J. Surg.* 2014 [48]	Can	Manitoba Cancer Registry Database, survey rectal cancer patient recruited by rectal cancer-treating surgeons who were identified by convenience sample	Patients diagnosed with adenocarcinoma of the colon or rectum, 1 January 2004 and 31 December 2006, English speaking patients, stage I–III disease, who had not yet undergone curative-intent surgery, but were scheduled to undergo curative-intent surgery
Monson J.T. et al. *Ann. Surg.* 2014 [49]	USA	Retrospective review of NCDB	Inclusion: patients diagnosed with rectal cancer, stage II and III, between 2006 and 2011, who underwent surgical resection, histology: adenocarcinoma, mucinous adenocarcinoma, signet rig cell carcinoma
Sankaranarayanan J. et al. *Expert. Rev. Pharmacoecon. Outcomes Res.* 2014 [50]	USA	National Cancer Registry	Inclusion: colorectal cancer patients, age 19+, between January 1998 and December 2003, diagnosed with colon cancer, rectosigmoid cancer, or rectal cancer Exclusions: ICD-O-3 codes C18.1, C21, C26.0, recurrence
Hines R. et al. *Am. J. Public Health* 2014 [51]	USA	Georgia Comprehensive Cancer Registry	Patients diagnosed with colon or rectal cancer, diagnosed between 2000 and 2007, age 45–85 years Exclusion: no prior lifetime cancer diagnosis (excluding non-melanoma skin cancer), multiple primary tumors, appendiceal tumors
Fleming S.T. et al. *J. Rural Health* 2014 [52]	USA	Cancer Registries of Kentucky, Ohio, Pennsylvania, and North Carolina, Center for Medicare and Medicaid Services Medicare Claims database	Inclusion: between 2005 and 2009, stage I–III colon cancer cases and stage III rectal cancer cases Exclusion: histology not adenocarcinoma, in situ cancers/stage missing, not first primary, multiple tumors, 66 < age > 80, metastases, no surgery
Helewa R.M. et al. *Dis. Colon Rectum* 2013 [53]	Can	Manitoba Cancer Registry, local recurrence information from chart review (paper and electronic charts)	Inclusion: diagnosed with stages I–III rectal adenocarcinoma, 2004–2006, treated at CancerCare Manitoba, had APR, pelvic exenteration, LAR, Hartmann’s, proctocolectomy, or trans-anal excisions Exclusions: rectosigmoid cancers treated as colon cancers, anal cancers, metastatic disease, unresectable disease at time of index operation, only polypectomy
Stewart D.B. et al. *Ann. Surg. Oncol.* 2013 [54]	USA	Pennsylvania Cancer Registry, linked to medical claims data provided by Highmark Inc. (insurance company)	Inclusion: rectal cancer, treated with surgical resection, 2004–2006 Exclusion: local excision, in situ carcinoma
Hines R.B. et al. *J. Rural Health* 2012 [55]	USA	SEER data from participating counties in Georgia (Atlanta Registry and Rural Georgia Registry)	Inclusion: patient diagnosed with colon/rectal cancer, first lifetime cancer diagnosis (excluding non-melanoma skin cancer), 1992–2007 Exclusion: appendix cancer
Sankaranarayanan J. et al. *Am. J. Manag. Care* 2010 [56]	USA	Nebraska Cancer Center Registry	Inclusion: patients age > 19, 1998–2003, diagnosed: colon cancer, rectosigmoid cancer, or rectal cancer, initial tumor Exclusion: carcinoid of appendix, anal canal carcinoma, or unspecified digestive organs, subsequent tumors if multiple
Esnaola N.F. et al. *Ann. Surg.* 2009 [57]	USA	South Carolina Central Cancer Registry	Inclusion: non-metastatic primary invasive colon and rectal cancer, 1996–2002 Exclusion: diagnosis at autopsy, metastatic disease, select morphology codes

## Appendix B

**Table A2:** MeSH search strategy used in the literature review, performed on 31 October 2024.

“((((((((((((“Rectal Neoplasms”[Mesh]) OR (“Rectal Neoplasm”[Title/Abstract])) OR (“Rectum Neoplasm”[Title/Abstract])) OR (“Rectal Tumor”[Title/Abstract])) OR (“Rectal Tumors”[Title/Abstract])) OR (“Cancer of Rectum”[Title/Abstract])) OR (“Rectal Cancer”[Title/Abstract])) OR (“Rectum Cancers”[Title/Abstract])) OR (“Rectal Cancers”[Title/Abstract])) OR (“Rectum Cancer”[Title/Abstract])) OR (“Cancer of the Rectum”[Title/Abstract])) OR (“Rectal Neoplasms”[Title/Abstract]))
AND
((((((rural)) OR (“Rural Health”[Mesh])) OR (“Rural Health Services”[Mesh])) OR (“Rural Population”[Mesh])) OR (“Hospitals, Rural”[Mesh])) OR (“non-urban”)”
